# HCC portal hypertension imaging score derived from CT predicts re-bleeding and mortality after acute variceal bleeding

**DOI:** 10.1186/s40644-024-00689-5

**Published:** 2024-03-28

**Authors:** Mingyuan Zhao, Binyue Zhang, Jianqiang Shi, Xiaoxian Tang, Hongxia Li, Shengwen Li, Yunfeng Yang, Yi Han, Rong Wang, Jian Xun, Kai Zhang, Xirun Wu, Jiang Zhao

**Affiliations:** 1 Department of Oncology, Shanxi Provincial People’s Hospital, Affiliated to Shanxi Medical University, Taiyuan, 030012 China; 2 Department of surgery, Shanxi Medical University, Taiyuan, 030001 China; 3 Department of Radiology, Shanxi Provincial People’s Hospital, Affiliated to Shanxi Medical University, Taiyuan, 030012 China; 4 Department of Hepatology, Shanxi Provincial People’s Hospital, Affiliated to Shanxi Medical University, 99 Shuang Ta street, Taiyuan, 030012 China; 5 Department of Hepatology, Taiyuan No.3 Hospital Affiliated to Shanxi Medical University, Taiyuan, 030012 China; 6 Department of Radiology, Taiyuan No.3 Hospital Affiliated to Shanxi Medical University, Taiyuan, 030012 China; 7 Department of Hepatology, Second Hospital of Shanxi Medical University, Taiyuan, 030001 China

**Keywords:** Variceal re-bleeding, Mortality, HCC, Liver cirrhosis, Specificity

## Abstract

**Background/Purpose:**

Risk factors for re-bleeding and death after acute variceal bleeding (AVB) in cirrhotic HCC patients are not fully understood.We aimed to (1) explore how the combination of high-risk esophageal varices, HCC status, and portal vein tumor thrombus (i.e., HCC Portal Hypertension Imaging Score [HCCPHTIS]) helps predict increased risk of variceal re-bleeding and mortality; (2) assess predictability and reproducibility of the identified variceal re-bleeding rules.

**Methods:**

This prospective study included 195 HCC patients with first-time AVB and liver cirrhosis, and conducted multivariable Cox regression analysis and Kaplan-Meier analysis. Receiver operating characteristic curve analysis was calculated to find the optimal sensitivity, specificity, and cutoff values of the variables. The reproducibility of the results obtained was verified in a different but related group of patients.

**Results:**

56 patients (28.7%) had re-bleeding within 6 weeks; HCCPHTIS was an independent risk factor for variceal re-bleeding after AVB (Odd ratio, 2.330; 95% confidence interval: 1.728–3.142, *p* < 0.001). The positive predictive value of HCCPHTIS cut off value > 3 was 66.2%, sensitivity 83.9%, and specificity 82.3%. HCCPHTIS area under the curve was higher than Child-Pugh score (89% vs. 75%, *p* < 0.001). 74(37.9%) death occurred within 6 weeks; HCCPHTIS > 4 was associated with increased risk of death within 6 weeks after AVB (*p* < 0.001).

**Conclusion:**

HCCPHTIS > 3 is a strong predictor of variceal re-bleeding within the first 6 weeks. However, patients with HCCPHTIS > 4 were at increased risk of death within 6 weeks.

**Supplementary Information:**

The online version contains supplementary material available at 10.1186/s40644-024-00689-5.

## Introduction

Hepatocellular carcinoma (HCC) is one of the most common tumors [1]. However, the most frightening event is acute variceal bleeding (AVB) in HCC patients with liver cirrhosis, especially variceal re-bleeding and higher mortality during 6 weeks after the first episode of AVB [2].When liver cirrhosis is coexists with HCC patients, several risk factors have been identified that are associated with variceal re-bleeding for an episode of AVB, including high-risk esophageal varices (EV), portal vein tumor thrombosis (PVTT), HCC state itself, model for end-stage liver disease (MELD), and the Child-Pugh score [3–6]. Upper endoscopy is considered the gold standard to determine the size of EV and ABV [4, 6–8], but this is painful for the patient. AVB and variceal re-bleeding are strongly associated with the portal hypertension (PHT). Elevated portal pressure is the driving force for variceal re-bleeding and the decompensation [1]. According to Baveno VI, VII conference, liver stiffness (LSM) and platelet count were found to be an accurate tool for PHT diagnosis in patients with liver cirrhosis, but not in HCC patients with liver cirrhosis [7, 8]. Combining LSM and platelet count did not evaluate the absence of high-risk EV in HCC patients, especially in patients with intermediate or advanced HCC [8]. Previous studies suggest that the presence of HCC may affect with elastometry results [9]. Furthermore, platelet count levels (in their absolute number) may be an increase in HCC [10]. HCC can impact LSM and platelet count levels with higher values than expected [9–12]. Therefore, in order to predict AVB and re-bleeding in HCC patients with liver cirrhosis, it is necessary to look for noninvasive and reproducible methods.

Depend on contrast-enhanced computed tomography (CT), HCC can be detected in liver cirrhosis [13, 14], as well as, the evaluation of PVTT and EV has been also collected in one examination [15–19].No additional costs would be incurred. Previous results suggest that a single imaging test may be used to provide an accurate estimation of portal hypertension [3–6]. However, risk factors for re-bleeding and mortality after AVB are incompletely understood in HCC patients with liver cirrhosis.

In recent years, the systematic treatment of liver cancer has made great progress [1, 20, 21].before making sound therapeutic decisions, the influence of multiple factors should be considered [22–24]. To be sure, the systematic treatment of HCC is limited due to the variceal bleeding. However, variceal re-bleeding is difficult to predict. Thus, it is a meaningful task to investigate the combination of high-risk esophageal varice, HCC state, and PVTT is helpful to predict the occurrence of variceal re-bleeding and mortality risk. Based on enhanced-CT, we exploratively assigned a different score to each imaging parameter. HCC portal hypertension imaging score (HCCPHTIS) was established in HCC patients with liver cirrhosis. We hypothesis that the HCCPHTIS can predict the increased risk of variceal re-bleeding and dying during 6-week after the first episode of AVB.

## Materials and methods

### Participants

This prospective observational study was performed in 3 centers in China (Shanxi Provincial People’s Hospital [Taiyuan], Second Hospital of Shanxi Medical University [Taiyuan], and Taiyuan No.3 Hospital [Taiyuan])0.195 HCC patients with liver cirrhosis who underwent both endoscope and enhanced-CT between January 2018 and January 2023 were eligible for analysis due to AVB. The diagnosis of liver cirrhosis was based on either histology or a combination of physical, laboratory, and radiological findings. Basis on typical imaging features on enhanced-CT, when necessary, with a biopsy, the diagnosis of HCC was established. the Barcelona Clinic Liver Cancer (BCLC) stages of HCC is defined by the patient’s general state, tumor state, liver function state, and available treatment options [25]. Liver disease severity was assessed according to the Child-Pugh score / classification, MELD. All patients with AVB were follow-up for at least 42 days, preferentially in clinic or else by telephone. Follow-up focused on final diagnosis, survival, variceal re-bleeding and hepatic decompensation at related variceal bleeding. The exclusion criteria: (1) age < 18 years; (2) transjugular intrahepatic portosystemic stent (TIPS) was used; (3) patients with non-variceal upper gastrointestinal bleeding. (4) overt hepatic encephalopathy before endoscopic examination and treatment.

### Endoscopy protocol

All patients underwent upper gastroesophageal endoscopy to assess the presence of AVB or re-bleeding. All endoscopies were performed with curative intent. AVB was treatment by 2 experienced endoscopists (Y.H., and Y.F.Y., two specialists in gastroenterology and hepatology, with 10 and 13 years of experience, respectively) who were blinded to the patient’s data. Ligation should be performed as needed. Participants will have follow-up endoscopic examination and treatment every 2 weeks until esophageal varices disappeared. If there was repeated variceal bleeding or new esophageal varices appear, the whole rigmarole starts over again. Whenever variceal bleeding was not obvious, final judgment was made with consensus among 2 endoscopists.

### CT protocol

All patients were examined with a CT scanner (Force, Siemens, Germany) using a dual source CT scanner and spiral CT scanner (Brilliance ICT, Philips, Holland). Enhanced CT was performed by one of two board-certified radiologists. A four-phase examination protocol of liver dynamic CT (unenhanced CT, arterial phase, portal-venous phase, and equilibrium phase) should be employed for the diagnosis of HCC. Scan parameter settings were as follows: tube voltage of 120 kV, with automatic tube current, a layer thickness of 5 mm and a 1.5 mm reconstruction interval.

### Imaging analysis

Two radiologists (J.Q.S., and X.X.T., with 25 and 30 years of experience with contrast-enhanced CT, respectively) independently reviewed the stored images and recorded the afore mentioned image features. They were blinded to the final diagnosis and laboratory results but was aware that these participants were at risk for HCC with liver cirrhosis. Imaging analysis was performed using a picture archiving and communication system (Siemens, Syngo. Via and Philips, IntelliSpace Portal) with adjustment of the optimal window width and level. In line with the previous literature [17–19], the imaging features of PVTT include an enhancement of contrast in the arterial phase and washout in the portal venous phase of the procedure. The presence of esophageal varices and the approximate size were observed by measuring the diameter of the largest observed varix in the portal venous phase. The 4-mm size criteria for large varices seen on CT was chosen as the high-risk varices based on Kim studies [15, 26].

#### Hepatocellular carcinoma portal hypertension imaging score (HCCPHTIS)

We introduced a new parameter (that is, the risk factors of portal hypertension in HCC patients with liver cirrhosis). HCCPHTIS include EV, solitary HCC, multifocal HCC, and PVTT.

On the basis of axial and coronal enhanced CT, we determined the following (Table 1):

1. A score of 1 or 0 was assigned if existence of EV was yes or no. Size of EV ≥ 4 mm was score of 2.
2. Size of solitary HCC ≤ 5 cm, or multifocal HCC ≤ 3 was score of 1.
3. Size of solitary HCC > 5 cm, or multifocal HCC > 3 was score of 2.
4. When PVTT was found, a score of 1 was assigned, but a score of 2 was assigned during tumor thrombus involving the main portal vein or involving the superior mesenteric vein [19].

**Table 1:** Definition and grading system for the three HCC portal hypertension imaging score parameters

Parameter	Points
EV quality score	
No presence of EV	0
Size of EV < 4 mm	1
Size of EV ≥ 4 mm	2
HCC state quality score	
Size of solitary HCC ≤ 5 cm,or multifocal HCC ≤ 3	1
Size of solitary HCC > 5 cm,or multifocal HCC > 3	2
PVTT quality score	
No presence of PVTT	0
No involving the main portal vein or the superior mesenteric vein	1
Involving the main portal vein or the superior mesenteric vein	2

EV,esophageal varices,HCC hepatocellular carcinoma;PVTT, portal vein tumor thrombus

The HCCPHTIS, ranging from 1 to 6 points, represented the sum of the above three parameters. We measured HCCPHTIS using CT as those parameters is easily obtainable, repeatable test, and noninvasive for estimation of occurrence of variceal re-bleeding and mortality in HCC patients with liver cirrhosis, and when liver lesions were diagnosed by CT, HCCPHTIS was also collected in one examination.

### End point

Primary end point: Occurrence of variceal re-bleeding after the first episode of AVB was assessed and recorded during 42 days of follow-up. Follow-up was the time from the first episode of AVB. The second end point was mortality, hepatic decompensation at related variceal bleeding. For all patients, the all-cause mortality data in 42 days follow up were obtained from hospitalization and outside hospital, whichever came first. Grade II, III or IV hepatic encephalopathy was assessed and scored according to West Haven criteria [27]. West Haven criteria: Overt hepatic encephalopathy is diagnosed by clinical criteria and can be graded. Grade II: lethargy or apathy, disorientation for time, obvious personality change, inappropriate behavior, dyspraxia, asterixis. Grade III: somnolence to semi-stupor, responsive to stimuli, confused, gross disorientation, bizarre behavior. Grade IV: coma. Acute-on-chronic liver failure was defined according to the EASL-CLIF consortium definition [28]. EASL-CLIF consortium definition: acute jaundice and coagulopathy, followed by ascites ± hepatic encephalopathy < 4 weeks in undiagnosed or diagnosed chronic liver disease, including cirrhosis.

### Statistical analysis

Continuous variables are given as mean value with standard deviation, categorical variables as numbers with percentages.

In order to identify independent predictors of the occurrence of variceal re-bleeding after the first episode of AVB during 6-week, univariable and multivariable Cox regression analysis were performed. Potential risk factors that were significant (*p* < 0.05) in the univariable Cox regression analysis were included in a multivariable Cox regression analysis to analyze the odds ratios (OR) and 95% confidence intervals (CI).

Receiver operating characteristic curves (ROC) were calculated, in order to find the best sensitivity, specificity, and cut off values of the variables (that is, Child-Pugh score, MELD, and HCCPHTIS) in variceal re-bleeding after the first episode of AVB. According to cumulative incidence function curve, mortality was also evaluated during follow-up.

The reproducibility of the obtained results was evaluated in another group of patients in a different, although related. For all analyses, a *p*-value < 0.05 was considered statistically significant. Statistical analyses were finished using the SPSS package for Windows (SPSS 25 Inc., Chicago, Illinois, USA) and MedCalc for Windows version 15.0 (MedCalc Software, Ostend, Belgium).

## Results

### Characteristics of patients

**Fig. 1:**
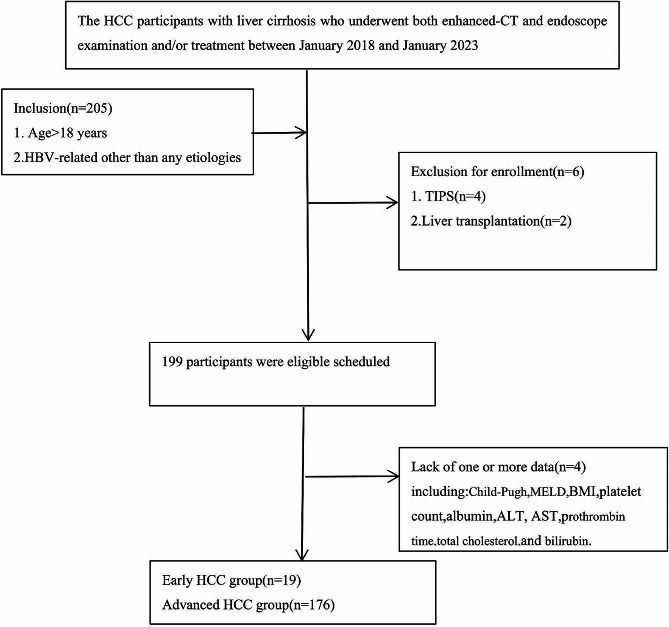
Flow diagram of study Of participants,195 were finally (included: early HCC group[*n* = 19] and advanced HCC group[*n* = 176]) ALT, alanine aminotransferase; AST, aspartate aminotransferase; BMI, body mass index; CT, computed tomography; HCC, hepatocellular carcinoma; MELD, model for end-stage liver disease; TIPS, transjugular intrahepatic portosystemic stent

A total of 205 HCC patients with liver cirrhosis were screened in the derivation cohort with HCCPHTIS. Ten patients were initially enrolled in the clinical trial, but subsequently removed from the study, six due to TIPS or liver transplantation after endoscopic therapy and the other for lack of one or more data. Eventually, 195 patients (mean age ± standard deviation, 60 years ± 11; age range, 31–83 years) were included in the study. In all patients, AVB was controlled by pharmacotherapy and endoscopic therapy. Nineteen patients (9.7%) were classified into the early HCC group based on our previously defined criteria; the remaining 176 (90.3%) patients were grouped into the advanced HCC group (Fig 1). Based on endoscopy, the majority of patients 153 (78.5%) were found to have esophageal varices alone, with 13(6.7%) of patients having only gastric varices, and 29(14.8%) of patients having both esophageal and gastric varices. The demographics and clinical characteristics of the study population are listed in Table 2. 60.0% (117 of 195) of patients were male, and 40.9% (78 of 195) were female. Liver function had an evaluation at the time of enrolment (Child-Pugh B 59.5% [116 of 195]) with a median MELD of 13.1 of the enrolled patients, and all HCC were HBV-related liver cirrhosis.

**Table 2:** Demographic and clinical characteristics

Variables	variceal bleeding (*n* = 195)
Age, y, mean ± SD	60 ± 11
Sex, male/ female, n (%)	117 (60.0) / 78 (40.0)
Child-Pugh class, A/B/C, n (%)	43 (22.1) / 116 (59.5) /36 (18.4)
MELD score	13.11(12.26,14.59)
BCLC stage, A/B/C/D, n (%)	24 (12.3) / 92 (47.2) /54 (27.7) / 25 (12.8)
Maximum tumor size, cm, mean ± SD	4.95 ± 1.78
Multifocal tumor, n (%)	67 (34.4)
Bilobar tumor involvement, n (%)	59 (30.2)
Extent of portal vein tumor thrombus Grade I/ Grade II/Grade III/Grade IV, n (%)	31(15.9) /30 (15.4) / 24 (12.3) /14 (7.2)
Prior curative resection or ablation, n (%)	63 (32.5%)
Size of EV ≥ 4 mm/Size of EV < 4 mm, n (%)	73 (37.4) / 77(39.5)
Prior overt hepatic encephalopathy, n (%)	26 (13.3)
Ascites, n (%)	182 (93.3)
ALT (IU/L)	26.7 (15.3–41.8)
AST (IU/L)	31.9 (23.0-51.2)
Prothrombin time, s	12.8 (11.8–14.6)
Serum albumin (g/l)	34.2 (29.8–38.7)
Total bilirubin (µmol/l)	29.1 (19.6–41.7)
Platelet count (*×*10^9^/mm^3^)	93.5 (69.8-110.2)
HCCPHTIS	3.0 (2.0–4.0)

*Data are numbers and data in parentheses are percentages; mean data are±standard deviation; Unless otherwise indicated, data in parentheses are interquartile range

ALT, alanine aminotransferase; AST, aspartate aminotransferase; BCLC, Barcelona clinic liver cancer; BMI, body mass index; EV, esophageal varices; HCC, hepatocellular carcinoma; HCCPHTIS; hepatocellular carcinoma portal hypertension imaging score; MELD, model for end-stage liver disease; PVTT, portal vein tumor thrombus

### Association between the HCCPHTIS and the occurrence of variceal rebleeding during follow-up

During follow-up in HCC patients with liver cirrhosis, 58% (113 of 195) HCC patients with complication related variceal bleeding occurred, including 50% (56 of 113) patient with variceal re-bleeding, 21% (24 of 113) with acute-on-chronic liver failure, 14% (16 of 113) with overt hepatic encephalopathy, 9% (10 of 113) with pneumonia, 4% (four of 113) with hepatorenal syndrome, and 2% (three of 113) with spontaneous bacterial peritonitis.

We studied the association between risk factors and the occurrence of variceal re-bleeding after the first episode of AVB during follow-up. Univariable Cox regression analyses were performed in order to find potential predictors of variceal re-bleeding. the MELD, Child-Pugh scores, prior overt hepatic encephalopathy, AST, platelet count, and HCCPHTIS were all significantly associated with 6-week variceal re-bleeding after the AVB episode (Table 3). Since these parameters (including: BCLC stage, maximum tumor size, multifocal tumor, the high-risk varices/the low-risk varices, PVTT) significantly association with HCCPHTIS, they were excluded in the multivariable Cox regression analysis to avoid the effect of collinearity. Multivariable Cox regression analysis showed HCCPHTIS (OR, 2.330, 95%CI 1.728–3.142, *p* < 0.001) was significant risk factors for the occurrence of variceal re-bleeding after the first episode of AVB (Table 3). It shows that HCCPHTIS was the independent risk of variceal re-bleeding after adjusting for prior overt hepatic encephalopathy, AST, platelet count, MELD, and Child-Pugh scores.

**Table 3:** Results of multivariate Cox regression analysis according to occurrence of re-bleeding

Variables	Univariate in variceal bleeding (*n* = 195)		Multivariat in variceal bleeding (*n* = 195)	
	Hazard Ratio (95% CI)	*P*-value	Hazard Ratio (95% CI)	*P*-value
Age (years)	0.988 (0.964–1.012)	0.318		
Gender (male vs. female)	1.679 (0.986–2.858)	0.056		
Child-Pugh score	1.300 (1.201–1.408)	< 0.001	1.080 (0.959–1.216)	0.202
MELD (score)	1.178 (1.107–1254)	< 0.001	0.978 (0.889–1.076)	0.646
BMI (kg/m^2^)	0.874 (0.749–1.020)	0.088		
BCLC stage, A/B/C/D, n (%)	3.013 (2.198–4.131)	< 0.001		
Maximum tumor size, cm, mean ± SD	1.132 (1.019–1.257)	0.021		
Multifocal tumor, n (%)	2.685 (1.581–4.561)	< 0.001		
Extent of portal vein tumor thrombus Grade I/ Grade II/ Grade III/Grade IV, n (%)	1.899(1.584–2.277)	< 0.001		
Size of EV ≥ 4 mm/Size of EV < 4 mm, n (%)	3.097 (1.962–4.887)	< 0.001		
Prior overt hepatic encephalopathy, n (%)	3.932 (2.237–6.913)	< 0.001	0.550 (0.238–1.274)	0.163
Ascites, n (%)	1.156 (0.418–3.199)	0.779		
ALT (IU/L)	1.002(0.996–1.008)	0.506		
AST (IU/L)	1.011 (1.003–1.020)	0.011	1.008 (0.997–1.018)	0.148
Prothrombin time (s)	0.910 (0.780 − 0.061)	0.228		
Serum albumin (g/l)	1.006 (0.966–1.048)	0.783		
Total bilirubin (µmol/l)	1.004 (0.998–1.009)	0.226		
Platelet count (*×*10^9^/mm^3^)	0.008 (1.002–1.015)	0.009	1.004 (0.997–1.010)	0.268
HCCPHTIS	2.277 (1.881–2.757)	< 0.001	2.330 (1.728–3.142)	< 0.001

ALT, alanine aminotransferase; AST, aspartate aminotransferase; BCLC, Barcelona clinic liver cancer; BMI, body mass index; CI, confidence interval; EV, esophageal varices; HCC, hepatocellular carcinoma; HCCPHTIS, hepatocellular carcinoma portal hypertension imaging score; MELD, model for end-stage liver disease; PVTT, portal vein tumor thrombus

### Association between the HCCPHTIS and mortality risk during follow-up

74/195 (37.9%) death occurred within 6-week. In the univariable analyses, the MELD, Child-Pugh scores, age, platelet count, AST, prior overt hepatic encephalopathy, and HCCPHTIS were all significantly associated with 6-week mortality. Multivariable Cox regression analysis showed HCCPHTIS (OR, 2.495, 95% CI 1.926–3.231, *P* < 0.001) was significant risk factors for the occurrence of variceal re-bleeding after the first episode of AVB (Supplemental Table 1). It was clear that HCCPHTIS was the independent risk of mortality after adjusting for age, prior overt hepatic encephalopathy, AST, platelet count, MELD, and Child-Pugh scores (Supplemental Table 1).

### Diagnostic performance of HCCPHTIS in identifying the occurrence of variceal re-bleeding and mortality risk

**Fig. 2:**
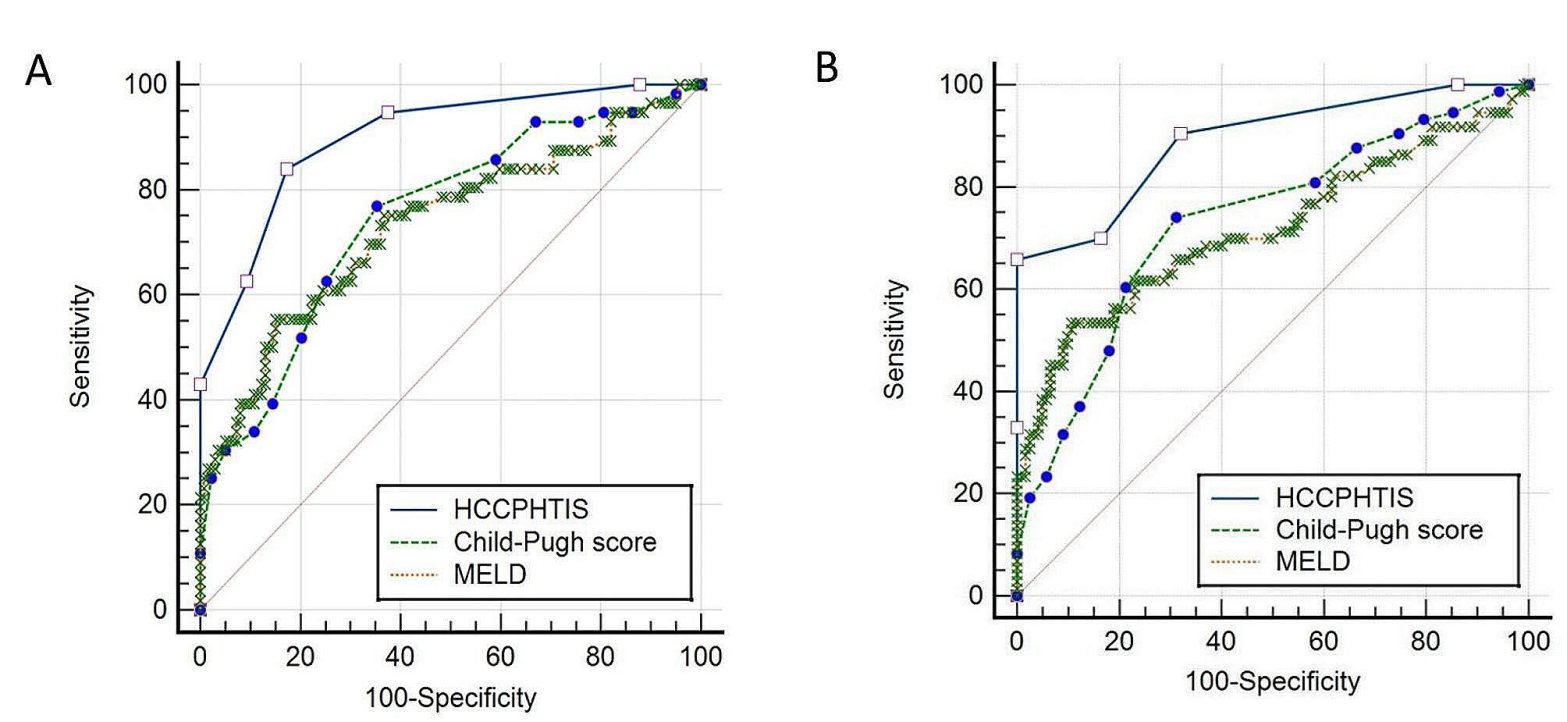
(**A**) The ROC curves for HCCPHTIS, MELD, and Child–Pugh scores for predicting 6-week re-bleeding of patients with variceal bleeding. AUC for HCCPHTIS, MELD and Child–Pugh scores were 0.89 (95%CI 0.853–0.946), 0.75 (95% CI: 0.670–0.825) and 0.73 (95% CI: 0.648–0.817), respectively. (**B**) The ROC curves for HCCPHTIS, MELD and Child–Pugh scores for predicting 6-week mortality of patients with variceal bleeding. AUC for HCCPHTIS, MELD and Child–Pugh scores were 0.89 (95% CI:0.844–0.937), 0.73 (95% CI: 0.659–0.807), and 0.72 (95% CI:0.636–0.798), respectively. AUC, area under the receiver operating characteristic; HCCPHTIS, hepatocellular carcinoma portal hypertension imaging score; MELD, model for end-stage liver disease

To identify potential image markers for the occurrence of variceal re-bleeding and mortality risk after the first episode of AVB in HCC patients with liver cirrhosis, we tested three parameters, including Child-Pugh score, MELD, and HCCPHTIS. The first, in the prediction of variceal re-bleeding, area under the ROC curve (AUC) for HCCPHTIS was numerically higher compared to either Child-Pugh score or MELD. There was statistically difference between HCCPHTIS (AUC 0.89, 95%CI 0.853–0.946) and Child-Pugh score (AUC 0.75, 95%CI 0.670–0.825) (*p* < 0.001). An HCCPHTIS achieved a 0.89 AUC compared to a 0.73 AUC (95%CI 0.648–0.817) (*p* < 0.001) of MELD (Fig. 2A). The second, when the prediction of the risk of death, The HCCPHTIS has still the higher AUC 0.88 (95%CI 0.844–0.937), superior to a Child-Pugh score (AUC 0.73, 95%CI 0.659–0.807) and MELD (AUC 0.72, 95%CI 0.636–0.798).There was statistically significant difference (HCCPHTIS vs. Child-Pugh score, *p* < 0.001; and HCCPHTIS vs. MELD, *p* < 0.001) (Fig. 2B). After this analysis, the HCCPHTIS and Child-Pugh score had a good diagnostic ability, with HCCPHTIS being the most promising one. The HCCPHTIS was chosen as parameter for selecting patients with a high risk of variceal re-bleeding and death at 6 weeks.

ROC analysis of HCCPHTIS for predicting the occurrence of variceal re-bleeding after the first ABV on the base-line was calculated (cut off > 3, sensitivity, specificity, positive predictive value and negative predictive value were 83.9%,82.7%,66.2% and 92.7%, respectively). However, for predicting the risk of death, a cut-off of > 4 (sensitivity 65.8%, specificity 100%, positive predictive value 100%, negative predictive value value 82.3%, respectively) was found at 6 weeks. After this study, the HCCPHTIS value > 3 was subsequently chosen as value for selecting patients with a high risk of variceal re-bleeding and HCCPHTIS > 4 was a criterion for risk of death at 6 weeks.

**Fig. 3:**
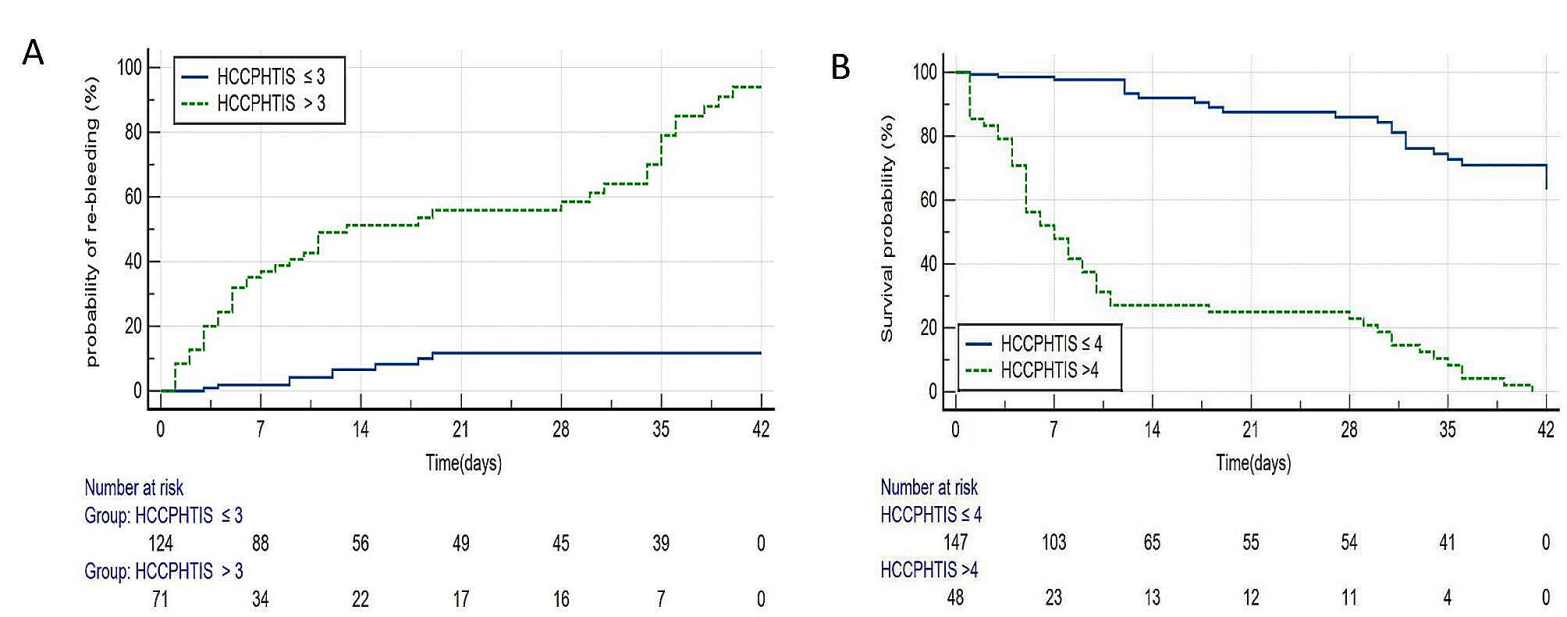
(**A**). Kaplan-Meier estimates of 6-week re-bleeding rate after acute variceal bleeding in patients stratified by HCCPHTIS (hepatocellular carcinoma portal hypertension imaging score). Figure 3 (**B**). Kaplan-Meier estimates of 6-week survival after acute variceal bleeding in patients stratified by HCCPHTIS (hepatocellular carcinoma portal hypertension imaging score)

Kaplan-Meier estimates according to HCCPHTIS cut-off value > 3 are shown in Fig. 3A. Variceal re-bleeding was 66.2% (47/71) in patients with HCCPHTIS > 3 and 7.3% (9/124) in patients with HCCPHTIS ≤ 3 (OR 4.51, 95% CI 4.17–21.83, *P* < 0.001) at 6 weeks. Stratification of patients according to HCCPHTIS (the higher HCCPHTIS > 4; the lower HCCPHTIS ≤ 4) revealed a significant increase in 6-week mortality after AVB between patients with higher compared with lower HCCPHTIS, (*p* < 0.001) in Fig. 3B.

### Validation of a HCCPHTIS as a predictor

There were 31 HCC patients with the first episode of AVB in this validation group. 22% (7 of 31) patients were Child-Pugh’s class A and 58% (17 of 31) were class B. After the first episode of AVB, 29.0% (9 of 31) of these HCC patients had variceal re-bleeding during 6-week of follow-up. Additional, characteristics of the group are displayed in Supplemental Table 2. The high diagnostic performance for identifying variceal re-bleeding in patients after the first episode of AVB could be confirmed in this validation group with an AUC of 0.90 (95%CI 0.78–0.99; *p* < 0.001) (Supplemental Fig. 1). Furthermore, the diagnostic accuracy of HCCPHTIS was affirmed. When the cut-off of > 3 was used, 83% (10 of 12) of the patients had variceal re-bleeding while 95% (18of 19) of patients with HCCPHTIS blow this cut off were free from variceal re-bleeding. according to HCCPHTIS > 4, 100% (11 of 11) of the patients had death.

## Discussion

Our study concerning the noninvasive diagnosis of variceal re-bleeding in HCC patients with liver cirrhosis were performed on a particular subgroup of patients (that is, patients who were AVB). We showed that HCCPHTIS can predict occurrence of variceal re-bleeding and mortality risk in HCC patients with liver cirrhosis during the 6-week after the first episode of AVB. To our knowledge, this method is the first study in this context in HCC patients with liver cirrhosis.

In our study, all HCC patients had liver cirrhosis, and nearly 36.4%(71/195) of them had the higher HCCPHTIS(that is, cut off > 3 ). The HCC patients with the higher HGGPHTIS had also the more existence of variceal re-bleeding compared with the lower HGGPHTIS (cut off ≤ 3 ) (*p* < 0.001) (Fig. 3A).We also noticed that a majority of dying HCC patients with liver cirrhosis had the higher HCCPHTIS (cut off > 4) (*p* < 0.001) (Fig. 3B).Therefore, according to the combination of EV, solitary HCC, multifocal HCC, and PVTT, it was advantageous for improving prognostic performance in HCC patients with liver cirrhosis during the 6-week after the first episode of AVB.

The most important findings of this study were that the higher HCCPHTIS (> 3 or > 4) can predict the risk of variceal re-bleeding and mortality after the first episode of AVB, providing an easy identification criterion for HCC patients with liver cirrhosis. The HCCPHTIS is composed of EV, HCC state, and PVTT, which are well-known risk factors for re-bleeding described in several studies [3–6]. These facts show that our exploring is consistent with the literature. Growing evidence has suggested that the HCC itself are considered adequate to elevate portal hypertension [3–5]. On the one hand, PHT is increased through the presence of arteriovenous shunting within the tumour and changes of liver architecture. Moreover, PVTT may have further increased portal pressure, thus exposing the patients to re-bleeding risk [2, 29]. On the other hand, esophageal varices in HCC with liver cirrhosis is phenotype of portal hypertension. Previous studies have used these manifestations to determine the presence of portal hypertension and severity, such as mortality, AVB, and re-bleeding [30–32]. Thus, on the basis of characteristic in HCC and cirrhosis, it’s possible to look for new judgment tools.

Some studies suggest that Child-Pugh classification and MELD help predict mortality, outcomes and other development of complications from HCC with liver cirrhosis, such as variceal bleeding, re-bleeding [1, 32, 33]. However, it still remains controversial which could reflect the prognosis more accurately in variceal bleeding and mortality [32, 33]. Our datum indicate that Child-Pugh score and MELD have the higher AUC on re-bleeding and mortality. However, HCCPHTIS has the highest AUC, superior to MLDE alone and Child-Pugh score. The higher HCCPHTIS (> 3 or > 4) is an accurate tool to predict variceal re-bleeding and mortality risk within 6-week after the first episode of AVB. It is worth noting that we further validated this innovative algorithm in an additional cohort with HCCPHTIS (> 3) and confirmed the robustness and wide range of its application during the first 6-week of follow-up. Our study showed that the predictiveness and reproducibility of the identified rules was assessed in appearance of variceal re-bleeding after the first episode of AVB during 6-week of follow-up, despite the limitations of the study design described below. Our study makes it even more concrete in clinical practice.

This study has some limitations. First, we observed that the assessment of HCCPHTIS was decided by differences of the parameter scores. However, it is not clear to PHT due to weight of every parameter in HCC with liver cirrhosis. we do not further provide clear information on the weight of each parameter in HCC with liver cirrhosis. This omission could limit the interpretability and reproducibility of the findings. We find that our information had further increased the prognostic weight of re-bleeding, therefore strengthening rather than weakening our results. Second, data regarding PVTT were available at entry only, and some patients actually may have developed PVTT during the follow-up period. Thus, it was not possible to evaluate the influence of de novo PVTT formation on patients’ prognosis. This is a problem to be solved further. Third, this study included a small number of patients with Child-Pugh class A, and C disease, potentially limiting the generalizability of the findings to broader patient populations. Further research including a more diverse patient group is necessary to validate the HCCPHTIS in various clinical contexts. Thus, further prospective, and multicenter studies that include more patients with Child-Pugh class. Finally, there was a small sample size. Therefore, its generalizability is not clear. According to the current literature, it is difficult to predict variceal re-bleeding when HCC exists with liver cirrhosis. However, our data highlight the importance of HCCPHTIS in predicting variceal re-bleeding in HCC patients with liver cirrhosis. it provides simple, easily applicable algorithms. With further validation, the results presented here could be adopted in identification of high-risk patients of variceal re-bleeding. It is anticipated that early and beneficiate endoscopic therapy or TIPS can be a great choice in prevent re-bleeding. Not unexpectedly, variceal re-bleeding prophylaxis is of paramount importance to improve survival. At the same time, we would like to know what happen to systemic therapy scenario in this patient population when variceal re-bleeding is under control.

## Conclusion

A high HCCPHTIS was found that there were predictive values in occurrence of variceal re-bleeding and increased risk of death within 6 weeks after an AVB episode. This study demonstrates that HCCPHTIS is an accurate, easy, and objective measure of PHT severity. Consequently, a HCCPHTIS of ≤ 3 and > 3 may be used in future studies to stratify patients into low risk and high risk for 6-week re-bleeding. When HCCPHTIS > 4, mortality risk is also evaluated.

### Electronic supplementary material

Below is the link to the electronic supplementary material.

Supplementary Material 1

Supplementary Material 2

Supplementary Material 3

## Data Availability

The datasets used and/or analysed during the current study are available from the corresponding author on reasonable request.

## Abbreviations

ALT: Alanine aminotransferase
AST: Aspartate aminotransferase
AVB: Acute variceal bleeding
BMI: Body mass index
CT: Computed tomography
EV: Esophageal varice
HCC: Hepatocellular carcinoma
PHT: Portal hypertension
PVTT: Portal vein tumor thrombus
MELD: Model for end-stage liver disease
