# Supplementary figures and images for: HCC portal hypertension imaging score derived from CT predicts re-bleeding and mortality after acute variceal bleeding
Source: Cancer Imaging. 2024 Mar 28;24:45. doi: 10.1186/s40644-024-00689-5 (PMC10976815; doi:10.1186/s40644-024-00689-5)

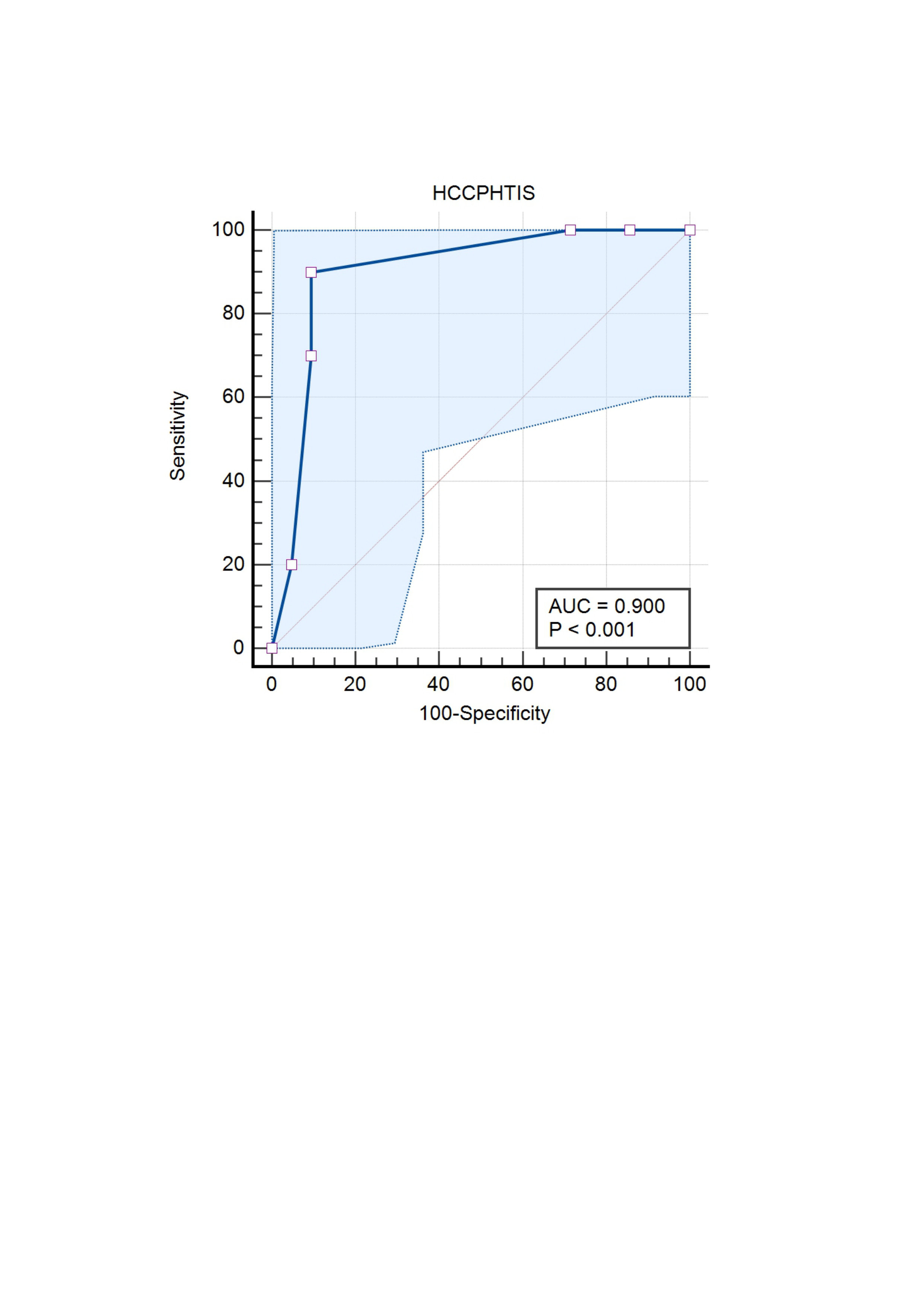

Supplement: Supplementary file 1 — Supplementary Material 1 [file 40644_2024_689_MOESM1_ESM.jpg]
