# Supplementary material for: HCC portal hypertension imaging score derived from CT predicts re-bleeding and mortality after acute variceal bleeding
Source: Cancer Imaging. 2024 Mar 28;24:45. doi: 10.1186/s40644-024-00689-5 (PMC10976815; doi:10.1186/s40644-024-00689-5)
Supplement: Supplementary file 2 — Supplementary Material 2 [file 40644_2024_689_MOESM2_ESM.docx]

Supplemental table 1.Results of multivariate Cox regression analysis according to risk of mortality

| **Predictor variables** | **Univariate in variceal bleeding(n=195)** |  | **Multivariat in variceal bleeding(n=195)** |  |
| --- | --- | --- | --- | --- |
|  | **Hazard Ratio(95%CI)** | ***P-*Value** | **Hazard Ratio(95%CI)** | ***P-*Value** |
| Age, y, mean ± SD | 0.974(0.953,0.996) | 0.018 | 0.973(0.950,0.997) | 0.029 |
| Sex,male/female,n (%) | 1.171(0.738,1.858) | 0.502 |  |  |
| Child-Pugh score | 1.285(1.198,1.379) | <0.001 | 1.080(0.976,1.195) | 0.138 |
| MELD score | 1.171(1.105,1.241) | <0.001 | 0.995(0.916,1.081) | 0.912 |
| BCLC stage,A/B/C/D,n(%) | 3.744(2.756,5.088) | <0.001 |  |  |
| BMI | 0.993(0.860,1.174) | 0.924 |  |  |
| Maximum tumor size, cm, mean ± SD | 1.577(1.405,1.771) | <0.001 |  |  |
| Multifocal tumor, n (%) | 0.304(0.175,0.526) | <0.001 |  |  |
| Extent of portal vein tumor thrombus, Grade I/ Grade II/ Grade III/Grade IV,n (%) | 1.944(1.639,2.305) | <0.001 |  |  |
| Size of EV≥ 4mm/Size of EV < 4mm, n (%) | 1.037(1.448,2.865) | <0.001 |  |  |
| Prior overt hepatic encephalopathy, n (%) | 3.769(2.233,6.361) | <0.001 | 1.398(0.182,1.869) | 0.021 |
| Ascites,n (%) | 1.001(0.434,1.309) | 0.988 |  |  |
| ALT(IU/L) | 1.002(0.997,1.008) | 0.455 |  |  |
| AST(IU/L) | 1.010(1.001,1.019) | 0.025 | 1.003(0.993,1.013) | 0.548 |
| Prothrombin time,s | 0.890(0.777,1.020) | 0.094 |  |  |
| Serum albumin(g/l) | 0.992(0.958,1.028) | 0.677 |  |  |
| Total bilirubin(µmol/l) | 1.002(0.958,1.028) | 0.410 |  |  |
| Platelet count(*×*10^9^/mm^3^) | 1.009(1.003,1.015) | 0.001 | 1.004(0.998,1.010) | 0.162 |
| HCCPHTIS score | 2.237(1.893,1.645) | <0.001 | 2.358(1.814,3.065) | <0.001 |

ALT,alanine aminotransferase;AST,aspartate aminotransferase;BCLC,Barcelona clinic liver cancer;BMI,body mass index;CI, confidence interval;EV,esophageal varices;HCC,hepatocellular carcinoma;HCCPHTIS,hepatocellular carcinoma portal hypertension imaging score;MELD,model for end-stage liver disease;PVTT,portal vein tumor thrombus.
