# Supplementary material for: HCC portal hypertension imaging score derived from CT predicts re-bleeding and mortality after acute variceal bleeding
Source: Cancer Imaging. 2024 Mar 28;24:45. doi: 10.1186/s40644-024-00689-5 (PMC10976815; doi:10.1186/s40644-024-00689-5)
Supplement: Supplementary file 3 — Supplementary Material 3 [file 40644_2024_689_MOESM3_ESM.docx]

Supplemental table 2characteristics of 31 HCC patients with variceal bleeding

| **Variables** | **variceal bleeding (n=31)** |
| --- | --- |
| Age, y, mean ± SD | 62±9 |
| Sex,male/female,n (%) | 25(80.6)/6(19.4) |
| Child-Pugh class,A/B/C,n (%) | 8(25.8)/18(58.1)/5(16.1) |
| MELD score | 13.55(12.97,14.68) |
| BCLC stage,A/B/C/D,n(%) | 5(16.1)/11(35.5)/13(41.9)/2(6.5) |
| Maximum tumor size, cm, mean ± SD | 4.8±4.2 |
| Multifocal tumor, n (%) | 9(29.0) |
| Bilobar tumor involvement, n (%) | 9(29.0) |
| Extent of portal vein tumor thrombus Grade I/ Grade II/ Grade III/Grade IV, n (%) | 4(12.9)/ 9(29.0)/6(19.4)/4(12.9) |
| Size of EV≥ 4mm/Size of EV < 4mm, n (%) | 12(38.7) / 10(32.3) |
| Prior overt hepatic encephalopathy, n (%) | 4(12.9) |
| Ascites,n (%) | 28(90.3) |
| ALT(IU/L) | 23.3(15.2-36.1) |
| AST(IU/L) | 37.4(27.1-59.0) |
| Prothrombin time,s | 12.7(12.2-15.2) |
| Serum albumin(g/l) | 33.9(29.9-39.4) |
| Total bilirubin(µmol/l) | 34.5(20.2-1.2) |
| Platelet count(*×*10^9^/mm^3^) | 88.6(49.6-120.5) |
| HCCPHTIS | 3.0(3.0-5.0) |

*Data are numbers and data in parentheses are percentages;mean data are±standard deviation;Unless otherwise indicated,data in parentheses are interquartile range.

ALT,alanine aminotransferase;AST,aspartate aminotransferase;BCLC,Barcelona clinic liver cancer;BMI,body mass index; EV,esophageal varices;HCC,hepatocellular carcinoma;HCCPHTIS;hepatocellular carcinoma portal hypertension imaging score; MELD,model for end-stage liver disease;PVTT,portal vein tumor thrombus.
